# Special properties of adult neurogenesis in the human hippocampus: Implications for its clinical applications

**DOI:** 10.1002/ctm2.1196

**Published:** 2023-02-05

**Authors:** Yi Zhou, Yijing Su, Guo‐li Ming, Hongjun Song

**Affiliations:** ^1^ Department of Neuroscience and Mahoney Institute for Neurosciences University of Pennsylvania Philadelphia Pennsylvania USA; ^2^ Department of Cell and Developmental Biology University of Pennsylvania Philadelphia Pennsylvania USA; ^3^ Institute for Regenerative Medicine University of Pennsylvania Philadelphia Pennsylvania USA; ^4^ Department of Psychiatry University of Pennsylvania Philadelphia Pennsylvania USA; ^5^ The Epigenetics Institute Perelman School of Medicine University of Pennsylvania Philadelphia Pennsylvania USA

**Keywords:** adult neurogenesis

1

Adult neurogenesis, the process of generating functional neurons from neural progenitors (Figure 1A), occurs throughout the lifetime in the hippocampus of almost all mammals examined, including humans.^1^, ^2^ Adult hippocampal neurogenesis plays critical roles in learning and memory, cognition, and affective behaviours, whereas its dysfunction has been associated with many neurological and psychiatric disorders, such as Alzheimer's disease (Figure 1B).^1^, ^2^ Studies in rodents have revealed distinct molecular, cellular, physiological, and neuronal circuitry properties of immature neurons (imNs) generated during adult neurogenesis compared to their mature counterparts (mNs), which are considered the foundation for the functional role of adult hippocampal neurogenesis.^2^ Limited knowledge of imNs in adult humans represents a major roadblock to harness their potential in clinical applications for brain disorders and regenerative medicine.

**FIGURE 1:**
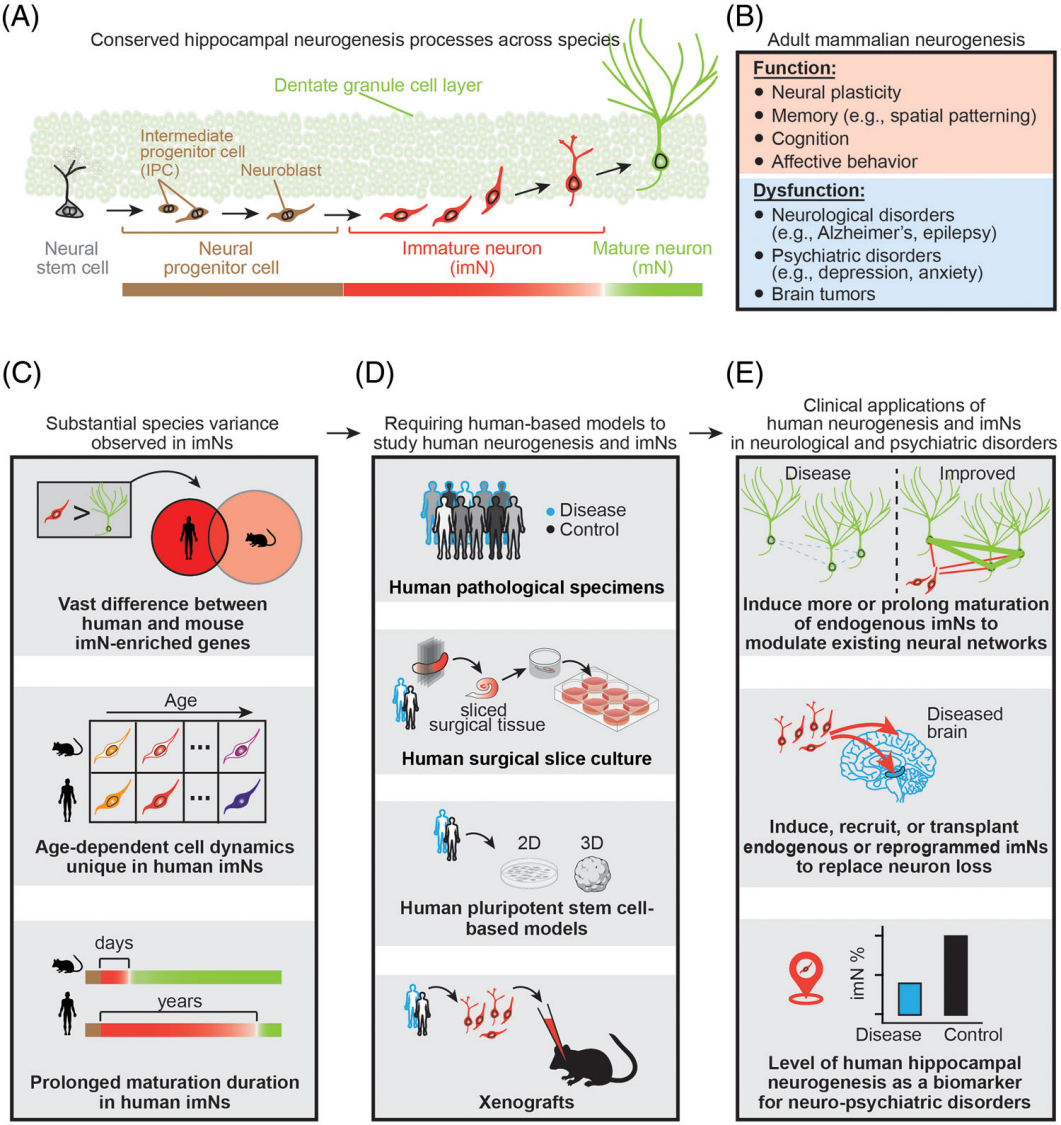
(A) Adult neurogenesis refers to the phenomenon that new neurons arising from adult neural stem cells persist throughout life in almost mammals examined, including humans. The dentate gyrus of the hippocampus is a major site for neurogenesis, and its overarching stepwise developmental processes are conserved across species. (B) The function of adult neurogenesis has been largely attributed to the unique properties of immature neurons (imNs) that are distinct from mature neurons (mNs). (C–E) Substantial species differences between imNs in humans and mice revealed by our published study^5^ highlight the necessity of using human‐based model systems to examine human‐specific cellular and molecular features, which will have broad implications in brain repair and regenerative medicine.

There are contradictory reports on the existence and abundance of newly generated neurons in the adult human hippocampus, mostly based on immunohistological analysis of one or two pre‐defined markers known in rodents.^3^, ^4^ Several single‐cell/single‐nucleus RNA sequencing (scRNA‐seq) analyses surveying the human hippocampus of different age groups did not identify a distinct imN cluster, including in the prenatal human hippocampus (summarized in Zhou et al.^5^). This is in sharp contrast to the clear sub‐clustering of immature progeny identified in the adult mouse hippocampal scRNA‐seq dataset,^6^ suggesting fundamental differences between species. Some studies^7^ performed correlation analysis by comparing human cells in their datasets to mouse imNs,^6^ assuming large transcriptomic resemblances between imNs of humans and mice. In our recent study,^5^ we leveraged the whole transcriptome scRNA‐seq to comprehensively characterize human imNs using a machine learning‐based analytic approach. We trained a classifier using our human infant hippocampus dataset and then used it to identify imNs in the human hippocampus across the lifespan. We found not only conserved immature features but also significant species differences between imNs in humans and mice in gene expression and temporal dynamics across ages (Figure 1C). We also performed immunohistological analysis of neuronal progenitor cells and developed an ex vivo culture system of human hippocampal surgical tissue to demonstrate the capacity for adult human hippocampal neurogenesis.^5^ Our results support continuous hippocampal neurogenesis in humans and suggest a model for retaining a large pool of imNs in the adult human hippocampus by low‐frequency *de novo* generation of neural progenitors and prolonged maturation of imNs (Figure 1A,C).^5^

Significant species differences revealed in our study^5^ (Figure 1C), and others^8^, ^9^ highlight limitations of using classic models, such as mice, to fully recapitulate features of human brain development or disorders, or to predict the impact of therapeutic treatment on human diseases. Not only did we observe substantial variance in imN‐enriched molecular signatures, we also found cell dynamics regarding transcriptomic shifts associated with age and potentially prolonged maturation in human imNs.^5^ Such cross‐species differences in various aspects have been widely observed in other brain regions (e.g., the developing neocortex) in humans in comparison with other species, such as mice and non‐human primates.^8^, ^9^ Therefore, direct analyses of human brain tissue or human‐based model systems are critical to examine mechanisms, pathologies, treatment strategies, and functions of human biological processes and diseases, including adult human neurogenesis. In recent years, procurement of high‐quality human brain specimens and advances in human pluripotent stem cell (hPSC)‐based model systems provide unprecedented opportunities to directly examine human brain development and diseases to understand their underlying cellular and molecular mechanisms (Figure 1D).^8^, ^9^ Despite the constraints of all emerging human models, such as the limited ability to study circuitry and behaviour, and an incomplete cell diversity recapitulated in the in vitro or ex vivo model systems, these technological advances, including neuropathological examination of patient specimens, patient surgical specimens for acute or organotypic slice culture, 2D and 3D hPSC‐derived models (e.g., brain organoids), and xenograft systems,^8^, ^9^ will bridge the gap between patient studies and animal models to revolutionize the study of brain development and diseases and therapeutic compound development in the next decade (Figure 1D).

Identifying imNs in the adult human tissue and revealing their molecular properties including human‐specific features and the capacity for being adult‐born opened a new, exciting avenue for exploring their potential clinical and translational applications (Figure 1E). Distinct properties of newborn imNs compared to their mature counterparts, which was previously shown in the rodent hippocampus,^2^ have been identified to be conserved in human imNs of different ages (Figure 1A).^5^ Here, we provide a few examples of the clinical potential once adult human neurogenesis and imNs are properly assessed and understood. One of the potential therapeutic applications would be to increase either the cell number or the duration of immature state of the highly plastic imNs, which modulate mature neuron firing, synchronization, and network oscillations,^2^ to impact the existing mature neural networks in neurological or psychiatric disorders, such as to excite dormant neuronal circuitry in degenerated brains (Figure 1E). Alternatively, imNs from endogenous neurogenic regions or produced by reprogramming may be used to replace lost neurons in the hippocampus or non‐neurogenic regions of diseased brains (Figure 1E). Moreover, studying the pathology and pathogenesis underlying adult human neurogenesis and imNs in neuropsychiatric disorders has important implications for understanding disease mechanisms. As neurogenesis and imNs in the adult human hippocampus are vulnerable to neurological and psychiatric disorders,^1^, ^2^, ^5^ their levels and gene expression patterns may serve as a biomarker and an indicator for human brain diseases, which can be quantitatively assessed at the single‐cell resolution by the emerging digitalized pathology technologies, such as spatially resolved transcriptomics (Figure 1E).

In summary, significant species variance was revealed by our study,^5^ which suggests challenges of studying the special properties of human hippocampal imNs and highlights the need to use human‐based systems for future investigations of their properties and clinical applications in diagnostics and treatment strategies.

## CONFLICT OF INTEREST STATEMENT

The authors declare no competing interests.
